# Dual chaotic encryption method for wireless communication privacy data based on deep learning

**DOI:** 10.1371/journal.pone.0341253

**Published:** 2026-06-23

**Authors:** Hongbo Yu

**Affiliations:** School of Communication and Electronic Engineering, Qiqihar University, Qiqihar, China; Maulana Abul Kalam Azad University of Technology West Bengal, INDIA

## Abstract

In wireless communication, the multipath effect and the time-varying channel due to mobility will directly lead to the key update cycle lagging far behind the channel change, which is difficult to effectively resist various malicious attacks and stealing behaviors, and affects the effect of privacy data protection in wireless communication. To this end, a deep learning-based dual chaos encryption method is proposed for wireless communication privacy data. Combining the chaotic characteristics of one-dimensional Logistic mapping and two-dimensional Henon mapping, the dual chaotic key is generated to extend the key space and improve the anti-attack ability; and the bidirectional long and short-term memory network (BiLSTM) is used to analyze the data such as key usage records, accurately predict the timing of the key updating, and generate a new key when anomalies are detected, and then distribute it securely. Taking the updated double chaotic key as input, the AES algorithm is used to realize wireless communication privacy data encryption through key expansion, initial round encryption, multiple rounds of iterative encryption and final round encryption, while the decryption process restores the plaintext by inverse operation. Experiments demonstrate that the method can effectively realize wireless communication privacy data encryption, and the security index can reach more than 0.94 in the face of different types of network attacks. It demonstrates that the proposed method can have the ability to resist all kinds of attacks and protect the security of private data.

## 1. Introduction

Wireless communication technology, as a key means of information transmission, has been deeply integrated into all levels of social life [1], and massive amounts of private data are frequently transmitted in open wireless channels in various fields ranging from daily mobile communication, telemedicine, smart home to industrial Internet of Things [2]. However, with the wide application of wireless communication networks, the security and privacy of communication data are facing unprecedented challenges [3]. On the one hand, the openness of wireless signals makes the data highly susceptible to eavesdropping, tampering, and malicious attacks during transmission, leading to the leakage of users’ sensitive information (e.g., personal identity, financial data, business secrets, etc.), which brings huge losses to individuals, enterprises, and even the state [4]; on the other hand, the rapid development of communication technology has led to an explosive growth in the volume of data, and the traditional encryption methods, in dealing with the massive amount of data, are face problems such as high computational complexity, low encryption efficiency, and difficulty in adapting to the complex and changing wireless communication environment [5]. In order to guarantee the secure transmission of wireless communication private data, resist various potential malicious attacks and information theft, and effectively protect users’ sensitive information, many scholars have conducted research on encryption of wireless communication private data [6]. For example, Hassan et al. [7] superimposed multi-user data in the power domain at the information sending end, utilizing the non-orthogonal characteristics of NOMA technology, legitimate user obtains the target signal through SIC decoding technology, and the eavesdropper cannot correctly separate the signals due to channel differences. At the same time, combined with the channel randomization security technology, the channel characteristics are used to generate dynamic keys in transmission, which ensures that only legitimate users can restore the information and realize information encryption. However, this method is prone to the phenomenon of decoding error propagation, i.e., legitimate users in the process of successive SIC decoding, if the decoding of the preceding sequence of user signals fails, it will lead to the accumulation of the BER of the subsequent signals, which in turn affects the accuracy of the data transmission. Mohammadi et al. [8] obfuscated the original data through fuzzy logic encryption algorithms to generate irreversible ciphertexts, and embedded an authentication digital watermark in the data using a robust hash-based watermarking method to embed the digital watermark with authentication function in the data to increase the data complexity. During transmission, the legitimate receiver decrypts the data through the shared fuzzy key and extracts the watermark to verify the integrity and authenticity of the source, thus enhancing data security. However, in the dynamic change scenario of network environment, the applicability of the original rules and functions will decrease, thus affecting the encryption effect. Shukla et al. [9] utilizes blockchain smart contracts to achieve automated key negotiation and distribution, ensuring the secure generation and updating of symmetric encryption keys, data is encrypted with the key of the current round before transmission, and the ciphertext is stored on the chain along with the records of the key usage time and access privileges, and the data is stored using the Blockchain’s tamperability prevents key leakage; at the same time, node identity is verified through the consensus mechanism to ensure that only authorized devices can access the key and decrypt the data, realizing the synergistic protection of data encryption and key security. Although the blockchain is tamperproof and can prevent key leakage, the ciphertext and key usage time, access rights and other records are stored on the blockchain, the attacker can infer the user’s power habits, device usage and other sensitive information by analyzing the data records on the blockchain, which in turn reduces the data security. Patil et al. [10] Achieving node authentication of mobile self-organizing networks through distributed collaborative mechanism with encrypted transmission. First, nodes dynamically elect trusted cluster heads using ant colony optimization algorithm and verify node identity through Byzantine fault tolerance mechanism; second, cluster heads generate lightweight symmetric session keys in real time based on network topology changes and distribute them to legitimate member nodes with the help of genetic algorithm. During data transmission, the sender encrypts the packet using the temporary key of the current path, and the receiver decrypts it through the shared key chain in the cluster, thus realizing the encrypted transmission of data. However, the symmetric session key in this method is highly dependent on the generation and management of the cluster head, and when the cluster head suffers from malicious attacks or moves out of the network, it is easy to cause key chain breakage and data decryption failure, thus affecting the reliability of data transmission. Karimov et al. [11] proposed a novel digital communication technology based on coherent chaotic data transmission method. Modulate the signal by changing the symmetry coefficients in the discrete chaotic model obtained by a special numerical integration method instead of the system parameters. Use the second-order self adjoint semi implicit integration method to obtain the discrete master and slave models of the considered chaotic oscillator. However, due to the limited accuracy of actual circuit components and the influence of noise, channel distortion, and other factors on chaotic carriers during transmission, the performance of the chaotic synchronization circuit at the receiving end may be reduced, and even chaotic synchronization may not be achieved. Ivan et al. [12] describe a novel modulation technique for chaotic communication systems based on a generalized explicit second-order Runge-Kutta method. The proposed modulation technique outperforms traditional parametric modulation methods in both coverage and noise immunity. The obtained results can be effectively applied to the design of advanced chaotic-based communication systems and can be used to enhance existing architectures. However, its performance is still limited by the accuracy of the numerical method itself. Korba et al. [13] generated two novel cascade structures—3D cubic sine and 2D cubic cat chaos parameter maps—based on a new parameter-varying cubic mapping. This enhances chaotic complexity, yielding highly random chaotic sequences and an enormous key space. To provide optimal security for image transmission, several novel methods for steganography and orthogonal amplitude modulation symbol encryption based on the new maps are introduced. However, in practical applications, the increase in key space may also bring challenges to key management and distribution. It is necessary to ensure the secure transmission and storage of keys to prevent communication security from being threatened due to key leakage.

Deep learning can automatically learn complex feature representations and pattern laws from a large amount of data by constructing a network structure with multiple hidden layers to realize tasks such as classification, regression and prediction of data [14]. Chaotic secure communication brings new ideas and solutions to the field of information security by virtue of its advantages such as sensitivity to initial conditions, random-like characteristics and wide bandwidth [15,16]. In this paper, we propose a double chaotic encryption method for wireless communication private data based on deep learning, which aims to integrate the learning ability of deep learning and the strong confusion property of chaotic system to construct a more efficient and secure encryption system, so as to improve the security of wireless communication private data.

## 2. Wireless communication privacy data double chaos encryption

### 2.1. Wireless communication privacy data double chaos key generation

Traditional key generation methods are usually based on a single chaotic system for key generation, which has a certain degree of randomness, but the key space is relatively limited and faces the problem of insufficient security, which is susceptible to cryptanalytic attacks, such as exhaustive attacks and differential attacks [17,18]. In order to enhance the complexity of the key as a way to adapt to the needs of the dynamic network environment, this paper combines Logistic mapping and Henon mapping. Logistic mapping is first utilized to generate the underlying chaotic sequences, which are then further transformed and obfuscated by Henon mapping to generate more complex and secure keys. This combination increases the complexity of the key and makes it more difficult for an attacker to deduce the key by analyzing the chaotic sequence. Although adaptive Henon mapping has the advantages of anti chaotic degradation and larger key space, it requires additional design of complex parameter adaptive adjustment mechanisms, which will significantly increase computational overhead. The traditional Henon mapping, combined with optimized Logistic mapping, can achieve a large key space that is sufficient to resist common attacks [19]. At the same time, combined with the real-time key update mechanism of BiLSTM, it can dynamically cope with the possible degradation problems of chaotic systems, achieving a better balance between security and computational efficiency.

Logistic mapping belongs to one-dimensional discrete chaotic system, which is characterized by sensitivity to initial conditions and unpredictable long-term behavior [20], and its mathematical model is:


$$\begin{array}{c} x_{n+1}=x_{n}\times \zeta \times (1-x_{n}) \end{array}$$ (1)

Where, ζ represents the control parameter, and $x_{n}$ represents the chaotic system state value at the n th iteration, which is the source of randomness for generating the chaotic key for wireless communication privacy data. When x∈[0,1], ζ is close to 4, the Logistic system is in chaotic state, and its generated sequence is random and ergodic.

Since Logistic mapping produces sequences with the limitation of uneven distribution, for this reason, its mathematical model is optimized and the optimized formula is:


$$\begin{array}{c} x_{n+1}=(1+\zeta )\left(1+\frac{\text{1}}{\zeta }\right)x_{n}(1-x_{n}{)}^{\zeta } \end{array}$$ (2)

Henon mapping is a two-dimensional discrete chaotic system with complex dynamical behavior as well as high dimensional chaos [21], and its mathematical model is:


$$\begin{array}{c} \left\{ \begin{array}{c} @l@{x^{\prime }}_{n+1}=1-\alpha \cdot \overset{\text{2}}{\underset{n}{x^{\prime }}}+y_{n}\\ y_{n+1}=\beta \cdot {x^{\prime }}_{n} \end{array}\right. \end{array}$$ (3)

In the formula, α and β represent the control parameters, when α=1.4 and β=0.3, the Henon mapping system is in chaotic state at this time, the behavior of the system becomes complex and sensitive to the initial conditions, such as attractor, traversal and other characteristics. $x^{\prime }$ represents the state variable after Henon mapping iteration, which is the state value for the next iteration, $y_{n}$ represents the state variables of the system at the first n iteration, which are related to each other, and their values are constantly updated by the iterative formula, outputting two sequences with chaotic characteristics.

The sequences generated by Logistic mapping and Henon mapping are modulo processed, and then the double chaotic key for wireless communication privacy data with good randomness and security can be obtained. The specific steps are described as follows:

(1) Initialize chaotic system parameters and initial values

Logistic mapping initializes: randomly select a initial value $x_{\text{0}}$ and control parameter ζ, $x_{\text{0}}\in \left[0,1\right]$, ζ∈[0,4].

Henon mapping initialization: randomly select two initial values ${x^{\prime }}_{\text{0}}$ and $y_{\text{0}}$, ${x^{\prime }}_{\text{0}},y_{\text{0}}\in \left[0,1\right]$, while making the control parameters α=1.4, β=0.3.

(2) Iterative chaotic system generates chaotic sequence

According to Eq. (2), iterate from the initial value $x_{\text{0}}$ to generate Logistic chaotic sequence $\left\{x_{n}\right\}$.

According to Eq. (3), two Henon chaotic sequences $\left\{{x^{\prime }}_{n}\right\}$ and $\left\{y_{n}\right\}$ are generated by iterating from the initial values ${x^{\prime }}_{\text{0}}$ and $y_{\text{0}}$.

(3) Chaotic sequence processing – mode fetching operations

In order to make the chaotic sequence more suitable as a key, the modulo operation is applied to the generated chaotic sequence.

The Logistic chaotic sequence $\left\{x_{n}\right\}$ is subjected to modulo operation to get a new sequence $\left\{k_{x_{n}}\right\}$, where $k_{x_{n}}=\left\lfloor x_{n}\times 10^{m}\right\rfloor mod\;N$, m is the scaling factor, which are positive integers used to control the range of values before modulo, and N is the range of values of the key (e.g., N=256, which generates an 8-bit binary key).

Using the same method, the Henon chaotic sequences $\left\{{x^{\prime }}_{n}\right\}$ and $\left\{y_{n}\right\}$ are processed by taking the mode to get the new sequences $\left\{k_{{x^{\prime }}_{n}}\right\}$ and $\left\{k_{y_{n}}\right\}$.

(4) Generation of double chaotic key

The Logistic sequence and Henon sequence after the modeling process are combined to generate the double chaotic key.

In this paper, the alternating combination is used to generate dual chaotic key $\left\{K_{i}\;\right\}$ for wireless communication privacy data, and the element $K_{i}$ in the key can be described as:


$$\begin{array}{c} K_{i}=\left\{ \begin{array}{cc} @ll@k_{x_{n},\left\lceil i/2\right\rceil }, & if\;i\;is\;odd\\ k_{{x^{\prime }}_{n},\left\lceil i/2\right\rceil }, & if\;i\;is\;even\;and\;\left\lceil i/2\right\rceil \leq \left\lceil M/2\right\rceil \\ k_{y_{n},\left\lceil i/2\right\rceil -\left\lceil N/2\right\rceil }, & otherwise \end{array}\right. \end{array}$$ (4)

Where, i=1,2,…,2M, M represent the key length.

### 2.2. Wireless communication privacy data key update

**Fig 1:**
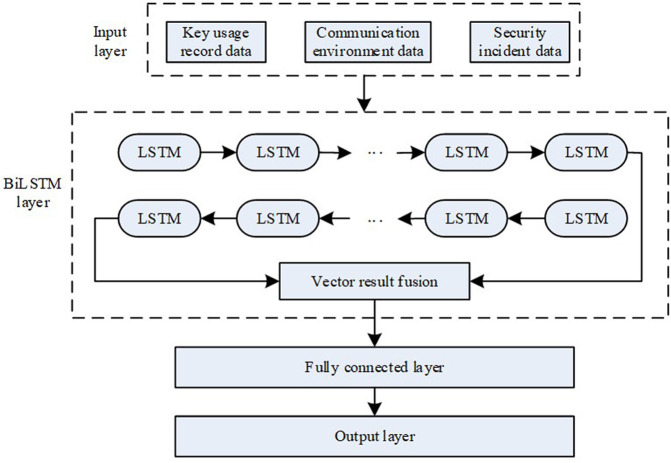
Wireless communication privacy data key update structure based on BiLSTM.

Due to the complexity and variability of the wireless communication environment, there are many potential security threats, and malicious attackers will try to crack the key to steal the private data [22]. Regularly updating the double chaotic key can increase the difficulty and cost of attackers, making it difficult for them to hold a valid key for a long time, so as to protect the security of the private data of wireless communication. LSTM, as a special kind of recurrent neural network (RNN), can remember the information of the key’s frequency of use and the updating interval over the past period of time, and by analyzing these temporal patterns, it can accurately predict when the key needs to be updated. LSTM as a widely used deep learning model, it has strong long sequence dependency processing ability, adaptive ability and generalization ability [23]. The model is able to learn and analyze the intrinsic patterns and potential trends of wireless communication traffic data by comprehensively considering multiple factors in the historical data, so as to provide an effective analytical tool for the detection of abnormal states of wireless communication traffic. However, the LSTM model does not fully utilize unidirectional information, and some information in long sequences is easily lost. For this reason, this paper adopts the bi-directional long and short-term memory network with the ability to capture positive and negative bi-directional information (BiLSTM) to promote the updating of the key in time and to ensure the security of the private data of wireless communication. The structure of wireless communication Ppivacy data key update based on BiLSTM is shown in Fig 1.

**Fig 2:**
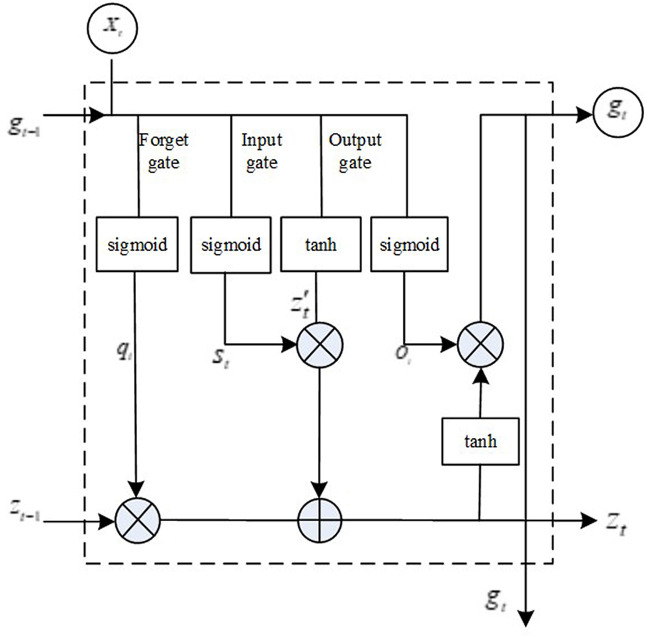
Structure of the LSTM unit module.

From Fig 1, the model consists of an input layer, BiLSTM, a fully connected layer, and an output layer. Among them:

(1) Input layer: The role of this layer is to receive a large amount of relevant data generated by the wireless communication system in the process of operation, mainly including key usage record data, communication environment data, security event data.
(2) BiLSTM layer: This layer is the core level of wireless communication traffic anomaly detection model, which learns and analyzes the input wireless communication related traffic data through forward and reverse LSTM modules respectively, and then fuses the hidden states of the two directions to obtain a more comprehensive representation of the wireless communication network traffic trend characteristics. A single LSTM contains three parts: input layer, hidden layer and output layer. Its implicit layer uses gated memory modules to replace ordinary input neurons. The structure of the LSTM unit module is shown in Fig 2.

In Fig 2, at the moment t, the inputs to the LSTM module mainly include: input data $X_{t}$, the state $g_{t-1}$ of the implicit layer output at the previous moment and the long-term state $z_{t-1}$ of the memory unit at the previous moment (the storage state of the historical traffic information at the previous moment, which represents the model’s memory of the long-term dependency of the wireless traffic data). The outputs of the LSTM module mainly include: the state $g_{t}$ of the implicit layer output at the moment t (which is a comprehensive representation of the current network traffic characteristics combined with historical memory, used for transmission to the next time step.) and the state $z_{t}$ of the memory unit (the comprehensive storage state of the current and previous wireless traffic information). The input gate in the LSTM unit module controls the degree of influence of $X_{t}$ on $z_{t}$, determining through the activation function how much information from the current input flow characteristics will be stored in the memory unit; the output gate controls the degree of influence of $z_{t}$ on $g_{t}$; and the forget gate controls and processes the historical flow data information in the memory unit. The related equation is described as:


$$\begin{array}{c} s_{t}=sigmoid(w_{s}⬝\left[g_{t-1},X_{t}\right]+b_{s}) \end{array}$$ (5)

$$\begin{array}{c} o_{t}=sigmoid(w_{o}⬝\left[g_{t-1},X_{t}\right]+b_{o}) \end{array}$$ (6)

$$\begin{array}{c} q_{t}=sigmoid(w_{q}⬝\left[g_{t-1},X_{t}\right]+b_{q}) \end{array}$$ (7)

Where, $s_{t}$, $w_{s}$ and $b_{s}$ represent the output results, weights and biases of the input gates in the model, $o_{t}$, $w_{o}$ and $b_{o}$ represent the output results, weights and biases of the output gates, and $q_{t}$, $w_{q}$ and $b_{q}$ represent the output results, weights and biases of the forgetting gates.

The outputs of the momentary LSTM unit module are $z_{t}$ and $g_{t}$ at the moment t, which are described by the formula:


$$\begin{array}{c} z_{t}=q_{t}⬝z_{t-1}+s_{t}⬝{z^{\prime }}_{t} \end{array}$$ (8)

Where, ${z^{\prime }}_{t}$ represents the candidate state of the memory cell at the moment t.

Based on $z_{t}$, the output gate leads to $g_{t}$, which is described as:


$$\begin{array}{c} g_{t}=o_{t}⬝tanh\left(z_{t}\right) \end{array}$$ (9)

After the bidirectional processing of network traffic data by BiLSTM, the hidden state sequences ${\vec{g}}_{t}=\left({\vec{g}}_{\text{1}},{\vec{g}}_{\text{2}}\cdots ,{\vec{g}}_{T}\right)$ and ${\overset{\leftarrow }{g}}_{t}=\left({\overset{\leftarrow }{g}}_{\text{1}},{\overset{\leftarrow }{g}}_{\text{2}},\cdots ,{\overset{\leftarrow }{g}}_{T}\right)$ in both directions are obtained, and the fusion of the two is implemented to obtain the wireless communication network traffic trend feature vector ${\hat{g}}_{t}$ corresponding to the current input network traffic information and integrating the preceding and following information, which is described in the following formula:


$$\begin{array}{c} {\hat{g}}_{t}=\left[{\vec{g}}_{t};{\overset{\leftarrow }{g}}_{t}\right] \end{array}$$ (10)

(3) Fully-connected layer: In this layer, the wireless communication network traffic trend feature output from BiLSTM layer is nonlinearly transformed and mapped from the high-dimensional space to the low-dimensional space, which is given by Eq. (11):

$$\begin{array}{c} H=f(W⬝{\hat{g}}_{t}+b_{H}) \end{array}$$ (11)

Where, H represents the output vector of the fully connected layer, W represents the weight matrix, and $b_{H}$ represents the bias.

(4) Output layer: This layer converts the output vector of the fully connected layer into a probability distribution by means of the softmax function. Each value in the probability distribution represents the probability of belonging to different categories of wireless communication network traffic (normal traffic, abnormal traffic). Based on this probability distribution determines whether the wireless communication network traffic is abnormal or not, which is described by the formula:

$$\begin{array}{c} Y_{i}=e^{h_{i}}/\overset{c}{\underset{j=1}{\sum }}e^{h_{j}} \end{array}$$ (12)

Where, $Y_{i}$ represents the probability value of abnormal wireless communication network traffic, c represents the traffic category, $h_{i}$ and $h_{j}$ represent the elements in the output vector H of the full connectivity layer.

When an anomaly in wireless communication traffic is detected, the key update mechanism is triggered, i.e., a new dual chaotic key is generated according to the method in subsection 2.1. A key negotiation protocol is used to securely distribute the newly generated key to both communication parties and to ensure that both parties update the key synchronously.

### 2.3. Encryption and decryption of private data in wireless communications

In order to safeguard the security of private data in wireless communication and prevent the data from being stolen, tampered with or maliciously utilized in the transmission process, the encryption and decryption of private data in wireless communication is processed by AES algorithm. AES is a widely used symmetric encryption algorithm, which has the characteristics of good security, high effect and easy to implement [24]. The basic idea of encryption and decryption is that after generating a specific double chaotic key, the sender uses the key and the AES algorithm to encrypt the plaintext information that needs to be transmitted, and the receiver uses the same key and the AES algorithm to decrypt the ciphertext.

In the AES encryption algorithm, the wireless communication privacy data to be encrypted first needs to be grouped to obtain the plaintext data block. In each encryption process, the result is abstractly described as a state which is represented as a two-dimensional byte array matrix. Its number of rows is fixed to 4 and the number of columns is determined by the length of the grouping and is the number of words contained in each group. In this algorithm, the relationship between its key length and the number of encryption rounds is shown in Table 1.

**Table 1:** Relationship between key length and the number of encryption rounds.

AES type	Key length/word	Number of encryption rounds
AES-128	4	10
AES-192	6	12
AES-256	8	14

**Fig 3:**
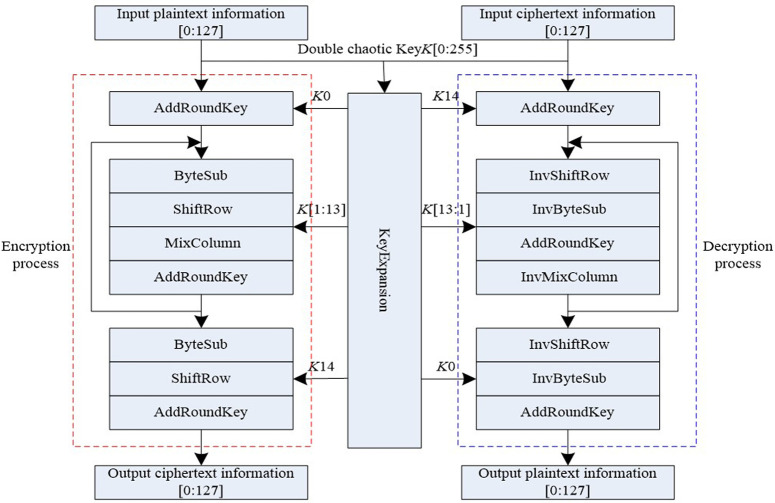
The process of encryption and decryption of private data in wireless communication.

In this paper, the AES −256 is used as an example to describe the wireless communication privacy data encryption and decryption process, as shown in Fig 3.

In Fig 3, the wireless communication privacy data encryption process is described as:

(1) Key Expansion: Input the updated wireless communication privacy data key K[0:255], and expand it into the round key used in multiple rounds by the key expansion algorithm. The expanded round key K0,K[1:13],K14,K[13:1] is used in each round of encryption and decryption respectively.
(2) Initial round Encryption: In this round, the round key plus operation is performed to introduce the key information into the plaintext.

The input 128-bit plaintext data [0:127] (wireless communication privacy data after grouped plaintext block), and the initial round key K0 after round key add (per bitwise different or) operation, to get the first round encrypted wireless communication privacy data state matrix. The formula for round key addition is described as:


$$\begin{array}{c} S\prime \left[i\right]\left[j\right]=S\left[i\right]\left[j\right]⊕K0\;\left[i\right]\left[j\right](0\leq i,j<4) \end{array}$$ (13)

Where, S[i][j] represents the byte value in the i row and j column in the plaintext block after grouping, S′[i][j] represents the byte value in the i row and j column in the state matrix after the initial round of encryption, ⊕ represents the bitwise different or operation.

(3) Multi-round iterative encryption: According to Table 1, for the AES-256 algorithm with 256-bit key, it is necessary to carry out 13 rounds of iteration (a total of 14 rounds of encryption). Each round contains the following operations:

S-transformation (byte substitution): its purpose is to increase the complexity of the data. This is done by nonlinearly substituting each byte in the state matrix of the wireless communication privacy data encrypted in the previous round using the S-box lookup table method. The formula for this is:


$$\begin{array}{c} \tilde{S}\left[i\right]\left[j\right]=S\_Box\left(S^{\prime }\left[i\right]\left[j\right]\right) \end{array}$$ (14)

Where, $\tilde{S}\left[i\right]\left[j\right]$ represents the byte value of after S-transformation, S_Box represents the fixed 16 × 16 lookup table.

Row shifting: Each row of the state matrix is shifted cyclically, specifically: row 0 is not shifted, row 1 is shifted left by 1 byte, row 2 is shifted left by 2 bytes, row 3 is shifted left by 3 bytes, and so on cyclically, which can achieve the effect of further confusing the data.

Column mixing: Each column in the wireless communication privacy data state matrix $\dot{S}$ after the row shift is linearly transformed to make the data more scattered, with the formula:


$$\begin{array}{c} \left[\begin{array}{c} @c@S^{\prime \prime }\left[0\right]\left[j\right]\\ S^{\prime \prime }\left[1\right]\left[j\right]\\ S^{\prime \prime }\left[2\right]\left[j\right]\\ S^{\prime \prime }\left[3\right]\left[j\right] \end{array}\right]=\left[\begin{array}{cccc} @cccc@02 & 03 & 01 & 01\\ 01 & 02 & 03 & 01\\ 01 & 01 & 02 & 03\\ 03 & 01 & 01 & 02 \end{array}\right]\cdot \left[\begin{array}{c} @c@\dot{S}\left[0\right]\left[j\right]\\ \dot{S}\left[1\right]\left[j\right]\\ \dot{S}\left[2\right]\left[j\right]\\ \dot{S}\left[3\right]\left[j\right] \end{array}\right] \end{array}$$ (15)

Where, $S^{\prime \prime }\left[i\right]\left[j\right]$ denotes the byte value of in the wireless communication privacy data state matrix after the column mixing operation.

Wheel key addition: when the wheel key K[1:13] of the current round is bitwise dissimilar to the state matrix to introduce the key information of the current round. Tthe formula is:


$$\begin{array}{c} \hat{S}\left[i\right]\left[j\right]=S^{\prime \prime }\left[i\right]\left[j\right]⊕K\left[1:13\right]\left[i\right]\left[j\right] \end{array}$$ (16)

(4) Final round encryption: Byte substitution, row shifting and round key addition (using the last round’s round key K14 to perform bitwise dissimilarity computation with the state matrix) operations are performed in the final round. The method of each link party operation is the same as the multi-round iteration.

After the final round, the encrypted 128-bit wireless communication privacy data ciphertext [0:127] is obtained.

The decryption process of wireless communication privacy data is, in essence, the inverse process of the encryption process, and the specific steps are as follows:

(1) Key Expansion: Use the same initial key K[0:255] as in the encryption process and perform key expansion to obtain the same round keys as in the encryption, which will be used in each round of decryption and in the reverse order of the encryption.
(2) Initial round decryption: The input AES-encrypted 128-bit ciphertext is keyed with the last round key K14 for round key addition. This step is a bitwise dissimilarity operation between the ciphertext and the round key to introduce the initial key information for decryption. The formula is:

$$\overset{͡}{S}\left[i\right]\;\left[j\right]\;=\overset{͝}{S}\left[i\right]\;\left[j\right]\;⊕K14\;\left[i\right]\;\left[j\right]\;\left(0\;\leq \;i,\;j\;<\;4\right)$$ (17)

Where, $\overset{͝}{S}\left[i\right]\;\left[j\right]$ represents the byte value in the ciphertext matrix and $\overset{͡}{S}\left[i\right]\;\left[j\right]$ represents the byte value after the initial round of decryption.

(3) Multi-round iterative decryption (13 rounds in total): each round of decryption contains the following operations:

Inverse Row Shift: Each row of the state matrix of the current round is shifted cyclically to the right, and the number of bits shifted in different rows is opposite to the cyclic left shift in encryption, i.e., row 0 is not shifted, row 1 is shifted to the right byte, row 2 is shifted to the right byte, row 3 is shifted to the right byte, and so on in a cyclic operation.

Inverse S-transform: use the inverse S-box to nonlinearly replace each byte in the current round’s state matrix, and restore the bytes that were replaced by the S-box during encryption.

Wheel Key Add: Perform bitwise dissimilarity operation between the wheel key K[1:13] of the current round and the state matrix to introduce the key information of the current round.

Inverse column mixing: Performs a linear transformation on each column of the state matrix, which is the inverse operation of the column mixing operation during encryption.

(4) Final round decryption: the final round decryption is similar to the multi-round iterative decryption, but omits the inverse column mixing operation, and only performs the inverse row shifting, inverse S-transformation, and round key addition, and the initial round key K0 is used in this round.

After the above operation, the decrypted 128-bit plaintext information can be obtained. It is shown that the above encryption method can effectively resist a variety of common attack methods. That is, the high sensitivity of the chaotic system to the initial conditions and parameters makes the encrypted data have random-like characteristics, which further improves the security of the data.

## 3. Experimental analysis

**Fig 4:**
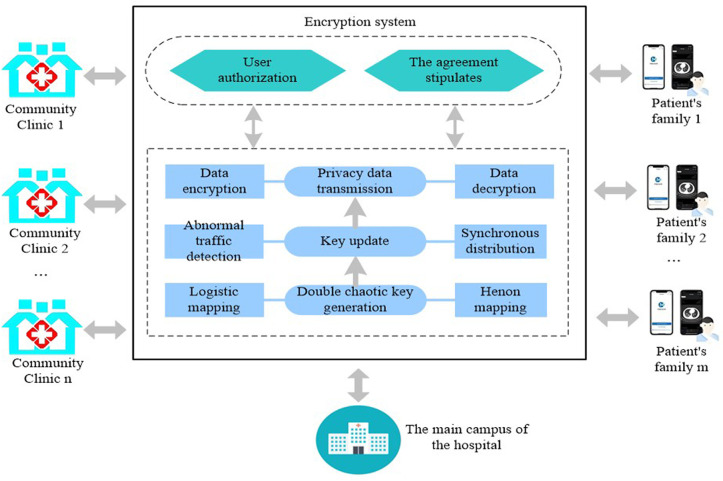
Shows the experimental platform built.

In order to analyze the effectiveness of the proposed deep learning-based wireless communication privacy data double chaotic encryption method in this paper, the wireless communication network applied to telemedicine is taken as the experimental object. The network covers multiple scenario nodes such as the hospital headquarters, community clinics and patients’ families, and adopts a hybrid networking mode of 4G/5G wireless network and Wi-Fi. Due to the business needs, a large amount of private data containing patients’ basic conditions, medical records, examinations and diagnostic results are transmitted every day. The experimental platform, as shown in Fig 4.

As can be seen from Fig 4, the experimental platform constructed connects multiple nodes such as hospital headquarters, community clinics, and patients’ families to the encryption system. Firstly, the base rules are constructed by user authorization and protocol provisions, and then the double chaotic key is generated by Logistic mapping and Henon mapping collaboratively, and the key update is triggered by abnormal traffic detection and then distributed to each node synchronously. During transmission, the private data is encrypted in the encryption system, and the ciphertext is restored at the receiving end by data decryption operation, thus realizing the secure transmission and processing of private data in telemedicine scenarios. During the experiment, the parameters of the algorithm are set as shown in Table 2.

**Table 2:** Main parameters of the experiment.

Name	Numerical value
The initial value of Logistic mapping $x_{\text{0}}$	0.8
The initial value of the Henon mapping ${x^{\prime }}_{\text{0}}$/$y_{\text{0}}$	0.4/0.4
Hidden layer of LSTM unit	4
The number of neurons in the hidden layer	80
Maximum number of iterations	100
Batch processing size	60
Learning rate	0.1

**Fig 5:**
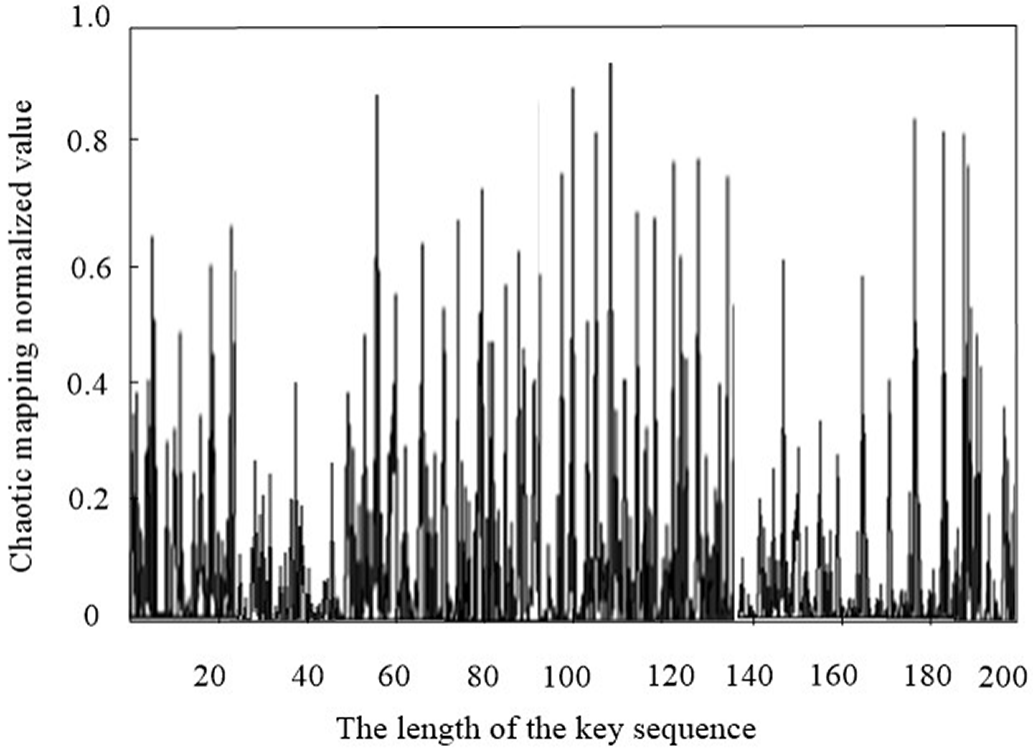
Shows the effect diagram of the double chaotic key generated by the method proposed in this paper.

In order to analyze the application effect of this paper’s method, a plaintext about the patient’s diagnosis and treatment results is constructed randomly and encrypted for transmission using this paper’s method. The experiment uses the chaotic characteristics of one-dimensional Logistic mapping and two-dimensional Henon mapping to generate the double chaotic key about the plaintext using the method of this paper, and The key is visualized with the help of Matlab software, and the results are shown in Fig 5.

Fig 5 shows the fluctuation characteristics and distribution range of normalized values of dual chaotic keys generated based on logistic mapping and Henon mapping. From the fluctuation situation, the key value has no obvious periodicity and regularity, showing highly irregular fluctuations; From the perspective of distribution range, the key values cover the range of 0–1.0, with uniform distribution and wide range. The above characteristics indicate that the generated dual chaotic key has good randomness and complexity, which can meet the high security encryption requirements of wireless communication privacy data.

**Fig 6:**
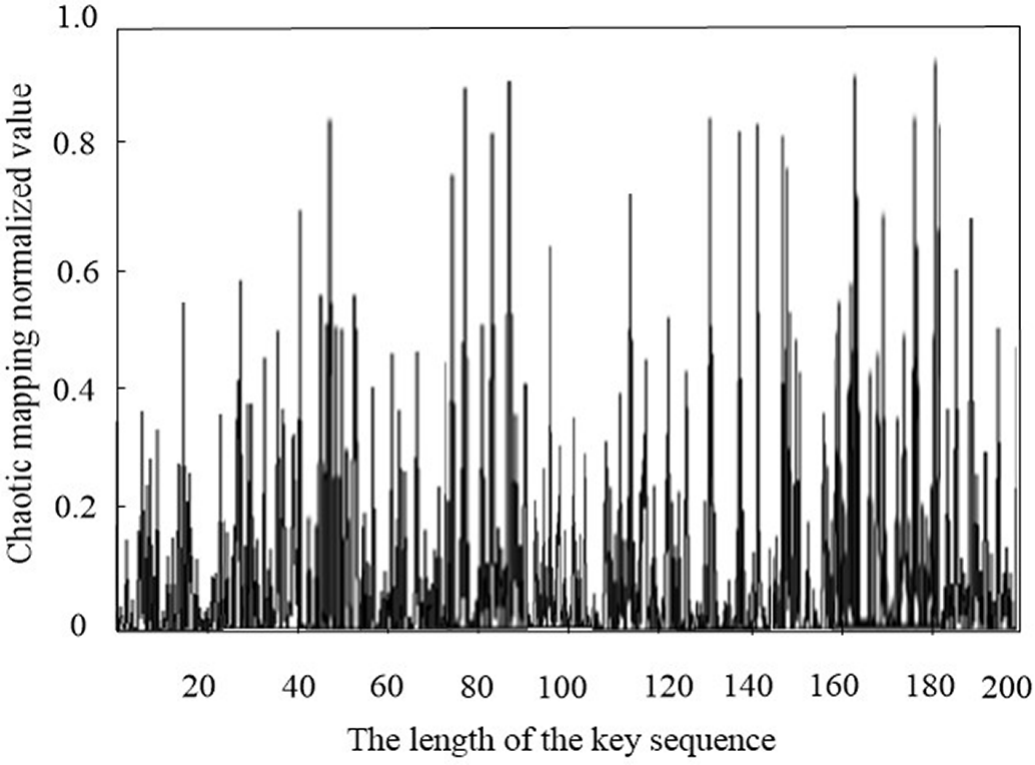
Shows the effect of the updated key.

The experiment constructs a wireless communication traffic anomaly detection model based on BiLSTM, and uses the model to detect the anomalous traffic in the network, and when the anomalous traffic is detected, the key is updated and the new key is sent to the two parties in the communication synchronously. The effect of the updated key is shown in Fig 6.

Fig 6 shows the fluctuation characteristics and distribution of the updated key. Compared with the pre update key, the updated key exhibits significant differences in fluctuation patterns and numerical distribution patterns while maintaining good randomness and wide distribution range. This indicates that the key update mechanism triggered by BiLSTM anomaly detection can generate dynamically changing new keys, effectively preventing attackers from cracking the encryption system by analyzing historical key patterns and ensuring the long-term security of wireless communication privacy data.

**Fig 7:**
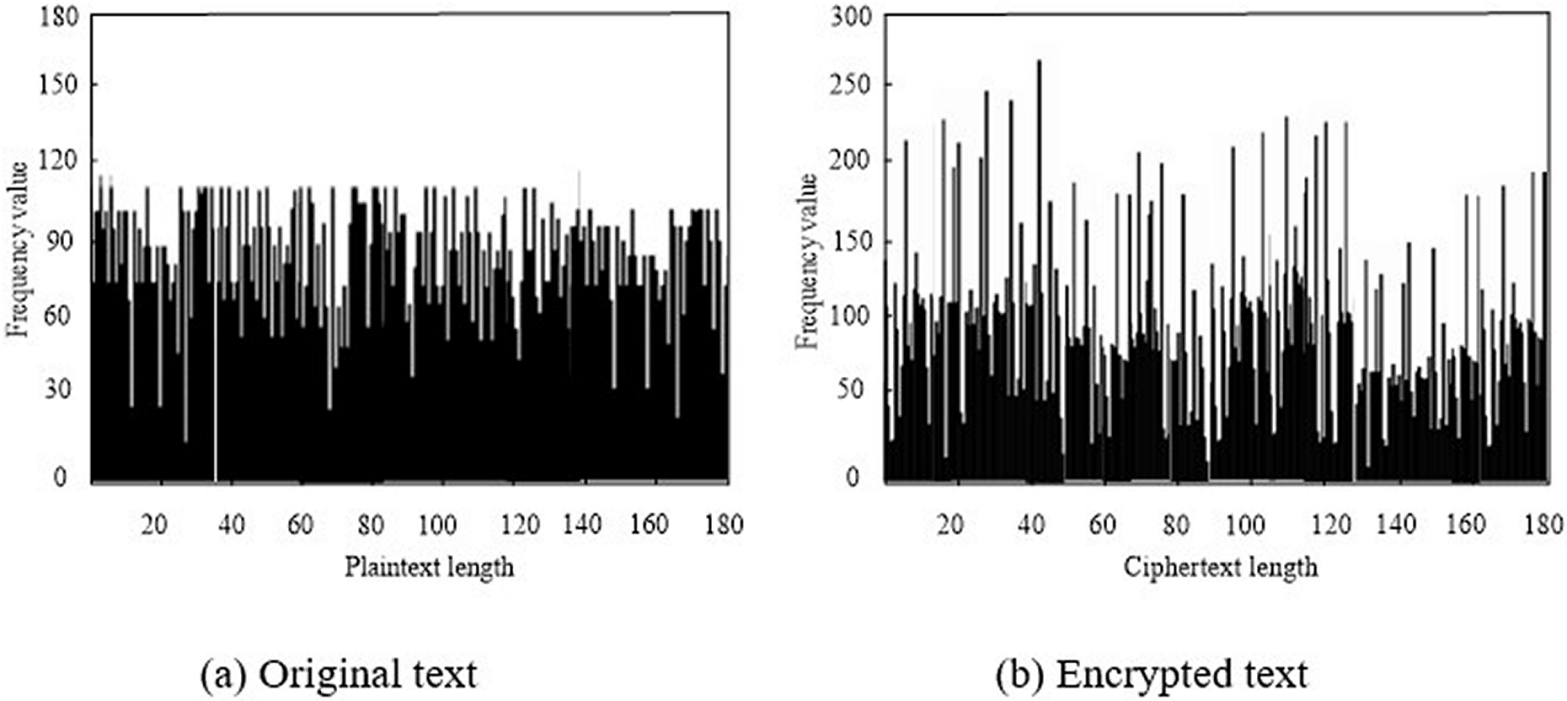
Comparison chart of Privacy Data encryption Effects in telemedicine networks.

The experiment realizes the telemedicine network private data encryption through AES algorithm with the double chaotic key of updated as input, after key expansion, initial round, multiple rounds of iteration and final round of encryption operation. The experiment analyzes the effect of encryption from the statistical point of view, and the results are shown in Fig 7.

From Fig 7, it can be seen that after the encryption of telemedicine network privacy data by this paper’s method, its statistical characteristics have changed greatly, and the distribution of the original text frequency values is relatively centralized and has a certain degree of regularity, while the distribution of the encrypted text frequency values is discrete and has a wider range, and the fluctuation is more complex. This shows that the method of this paper can significantly change the data characteristics, effectively confuse the original laws of privacy data, enhance the ability to resist network attacks, and safeguard the security of telemedicine privacy data.

**Fig 8:**
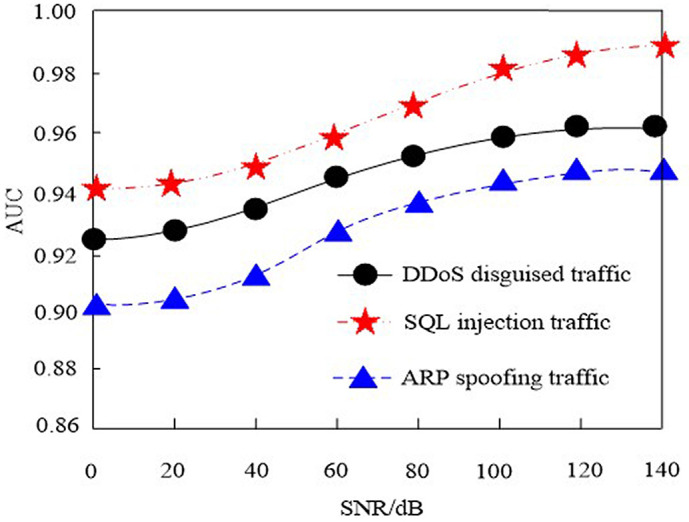
AUC situation of the BiLSTM model.

For the method of this paper, the anomaly detection of wireless communication network traffic is crucial, which is directly related to the timeliness of key update. For this reason, the experiments use the AUC value indicator to test the detection performance of the wireless communication traffic anomaly detection model constructed in this paper based on BiLSTM under the injection of DDoS camouflage traffic, SQL injection traffic, and ARP spoofing traffic. AUC, as an indicator that can reflect the classification ability and generalization performance of the detection model. The closer it is to 1, the stronger the model’s ability to distinguish between abnormal traffic and normal traffic, and the better the detection performance. After testing, the results are shown in Fig 8.

As can be seen from Fig 8, with the increase of SNR, the BiLSTM detection model improves its AUC value in the face of three different types of anomalous traffic, namely, DDoS camouflage traffic, SQL injection traffic, and ARP spoofing traffic, with the highest value reaching 0.99. In the environment of low SNR, the AUC value also reaches over 0.90. It further shows that the BiLSTM model has a strong ability to recognize all kinds of abnormal traffic, and with the increase of signal-to-noise ratio, its ability to distinguish between abnormal and normal traffic is increasing and strong.

**Fig 9:**
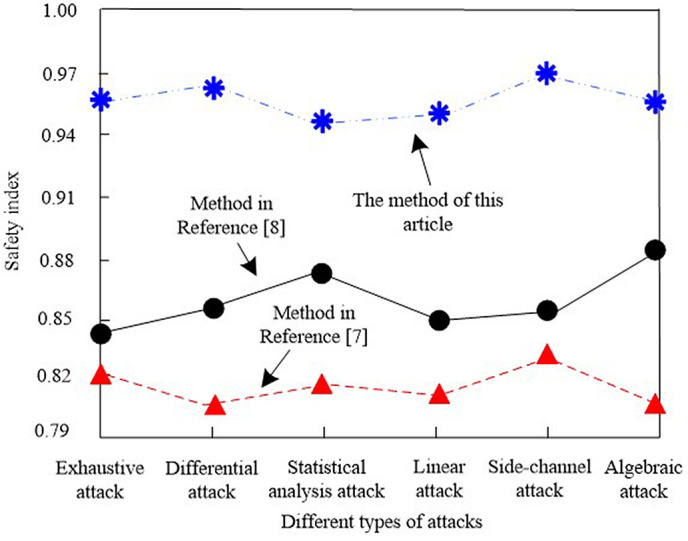
Security index situations under different attacks.

Security index is a comprehensive evaluation index that can be used to measure the security performance of the encryption method in the face of potential network threats and attacks, and the larger the security index, the better the encryption method performs in multiple security attributes such as confidentiality, integrity, availability, etc., and the better its ability to resist network attacks. For this reason, experiments were conducted to consider the security performance of this paper’s method using this index, and compared and analyzed with NOMA-based PLC network cooperative confidentiality method proposed in literature [7] and fuzzy encryption-based watermarking hybrid secure data transmission method proposed in literature [8], and the results obtained from the test, as shown in Fig 9.

From Fig 9, it can be seen that the security index of this paper’s method can reach more than 0.94 when facing different types of network attacks, which is significantly higher than that of the methods proposed in literature [7] and literature [8]. This shows that the method in this paper has stronger security protection ability and robustness, can more effectively resist diverse network threats, and can provide more reliable security for private data encryption scenarios.

To further verify the performance advantages of the proposed method, fractional order chaotic system encryption method and single logistic mapping encryption method were selected as comparison objects, and quantitative comparisons were made from three dimensions: key space size, encryption efficiency, and anti attack performance. The results are shown in Table 3.

**Table 3:** Performance comparison results of different chaos encryption methods.

Encryption method	Key space size Encryption	Efficiency/(s/GB)	Safety index
Encryption of fractional order chaotic systems	10¹²⁸	89.6	0.87
Single Logistic Mapping Encryption	10⁶⁴	65.3	0.79
The proposed encryption method	10^256^	52.1	0.96

From the data in Table 3, the proposed encryption method shows significant advantages in all three core performance indicators. In terms of key space, the proposed method’s 10^256^ far exceeds the 10^128^ of fractional order chaotic system encryption and the 10^64^ of single logistic mapping encryption, greatly improving the ability to resist exhaustive attacks; In terms of encryption efficiency, the proposed method takes 52.1 seconds to process 1GB of data, achieving higher real-time performance; In terms of anti attack performance, the proposed method has a security index of 0.96 and better protection effect. Overall, the proposed method achieves a better balance between security, efficiency, and resistance to attacks, with outstanding comprehensive performance.

## 4. Conclusion

The breakthroughs of artificial intelligence and other technologies have gradually exposed the security risks of traditional encryption technology in the face of increasingly complex network attacks and computational power enhancement, and it is urgent to seek more efficient and reliable confidential communication technology. In this paper, we propose a deep learning-based double chaotic encryption method for wireless communication private data, which realizes efficient encryption and secure transmission of wireless communication private data by organically combining the deep learning model with the chaotic system. The feasibility and effectiveness of the proposed technique are proved through experiments, which provides solid technical support for the secure construction of wireless communication private data. Future research will focus on further optimizing the lightweight design of the deep learning model to reduce the consumption of computational resources in the encryption process; exploring the fusion mechanism of multimodal chaotic systems to enhance the robustness of the encryption algorithm; and at the same time, combining with the techniques of federated learning and other technologies to achieve the co-evolution of the encryption model and the dynamic updating under the premise of safeguarding the privacy of the data, so that it can provide a more solid technical support for the protection of the privacy of wireless communications.
